# The impact of moral leadership on physical education teachers’ innovation behavior: The role of identification with leader and psychological safety

**DOI:** 10.3389/fpsyg.2022.1030245

**Published:** 2022-12-21

**Authors:** Jineng Chen, Wei Zheng, Binbin Jiang

**Affiliations:** ^1^Graduate School, St. Paul University Philippines, Tuguegarao, Philippines; ^2^School of Physical Education and Health, Sanming University, Sanming, China; ^3^School of Education, Quanzhou Vocational and Technical University, Quanzhou, China

**Keywords:** physical education, innovation behavior, moral leadership, psychological safety, identification with the leader

## Abstract

With the growth of people’s health needs and the impact of the COVID-19 pandemic, it is an inevitable trend to promote innovation behaviors of physical education (PE) teachers to innovate traditional physical education and adapt to national needs of sustainable development in the sports industry. Considering that moral leadership can promote innovation behavior of individuals through psychological factors, this study defines the types of innovation behavior, and from the perspective of psychological safety and identifying with leaders, discusses the impact of moral leadership on individuals’ innovation behavior by using hierarchical multivariate regression analysis, which provides inspiration for schools to strengthen the innovation behavior of physical education teachers. In this study, 327 questionnaires were distributed to PE teachers in Chinese provinces and 287 valid questionnaires were collected. The analysis of the collected data was performed with the help of the SPSSAU data analysis platform. The following conclusions were drawn: First, moral leadership has a significant positive impact on the psychological safety and internal and external innovation of physical education teachers. Secondly, moral leadership influences employees’ innovation behavior through psychological safety, and plays a part of intermediary role between moral leadership and internal and external innovation behavior; Third, by comparing the two impact mechanisms of innovation behavior, we found that moral leadership encourages employees to produce more external innovation behavior through psychological safety; Finally, strong leadership identity plays a positive role in regulating the relationship between moral leadership and innovation behavior.

## Introduction

The sudden outbreak of the COVID-19 epidemic in 2020 has had a huge impact on human life, quickly triggering people’s deep thinking and heated discussions on the autoimmune system, and the demand for physical exercise in pursuit of health has rapidly increased. Other innovation behaviors, such as technology innovation and platform innovation, have an impact on the optimization of human’s external living environment, such as bringing a stable and wonderful environment. While PE teachers’ innovation behavior can directly affect and satisfy individuals’ health exercise needs caused by the internal motivation of physical fitness improvement. Now that the influence of the external environment is hindering the traditional behavior of physical exercise, scholars have attracted a lot of attention to PE innovation. More and more countries are focusing on the development of the sports industry and the cultivation of relevant sports service personnel. For example, the 14th Five Year Plan in China seeks to strengthen the construction of a high-level physical education (PE) talent team and create a career development plan for them; To this end, China issued the Education Modernization 2035 plan in 2019, emphasizing the creation of “a high-quality professional and creative teacher team,” as “innovation teachers” have become an important feature of modern education. In 2021, the new curriculum reform including basic PE was fully launched. At the same time, the United States has promotes sports activities suitable for public participation, and has issued policy documents on the construction of mass sports, teachers and venue facilities in various periods, with notable policy effects (Riegelman and Garr, 2011). In addition, Germany has a clear concept in the development of the sports industry and the training of sports talents, vigorously supports the construction of sports clubs, and strives to create differentiated sports services to meet the different sports needs of the public, increases the public sports participation rate and sports consumption, and finally drives the rapid development of the sports industry (Breuer et al., 2015). PE teachers are a source of vitality in physical education innovation and the main force behind sports industry implementation. Therefore, ensuring that PE teachers do their work better, provide social sports services, and promote their innovation behavior is key to modernizing physical education, which has important practical significance.

Previous research on participants of PE innovation focused on the roles of government and market investor (Kanario, 2017), while research on PE talents was ignored. Part of the research on the antecedents that affect PE field’ innovation behavior focuses on preservice education content (Rottmann and Ratto, 2018), individuals’ sense of mission and responsibility (Ellison, 2022), and negative psychology, including burnout and occupational stress (Benevene et al., 2020). At the same time, many scholars also put forward that individual motivation promotes their innovation behavior (Nikiforos et al., 2020). When PE teachers have a strong incentive to renew old teaching ideas, they push the reform of physical education curriculum, and other innovation acts. In addition, some scholars have focused on the impact of the organizational innovation climate on the innovation behavior of PE teachers. Other factors, such as their leadership style and innovative activities, are relatively scarce and should be further explored. According to social cognitive theory, individual innovation behavior is a function that includes individual and environmental factors (Amabile, 1983). As the innovation process of physical education teachers is complex, competitive and constantly changing, they need the support of leaders, recognize and tolerate the contradictions and tensions in the innovation process (Hunter et al., 2011). Therefore, leaders are the key to realizing individual innovation behavior (Zhou and Hoever, 2014), and their specific leadership style affects the degree of employees’ innovation behavior in the workplace (Gong et al., 2009); Moral leadership touches the core of the overall progress and development of a school (Reed, 2004). It is an essential driving force for the continuous expansion and innovation of physical education. It has achieved remarkable results by encouraging PE teachers to carry out more extensive innovation behaviors. Many colleges and universities in China have shown outstanding performance in the field of traditional physical education innovation. Under the moral support of the school for sports innovation and the advocacy of undertaking national sports projects, PE teachers participate in seminars on physical education, so that they have a lot of inspiration and flexible time for open teaching. At the same time, the innovation behavior about internal teaching also allows teachers to creatively lead students to participate in the incubation project of the sports industry. Thus the internal innovation behavior was extended to the external innovation behavior of university-industry cooperation, such as developing teaching projects with ethnic minority style sports, which aroused great interest of students and widespread concern of society. Therefore, it is necessary to comprehensively consider the common influence of organizational leadership style, individual psychological safety and leadership cognition in the process of individual innovation. PE teachers are becoming an important source of sports talent reserve in the sports industry and the major practitioners in the sustainable development of physical education (Froberg and Lundvall, 2022). The innovation behavior of teachers involves not only the internal innovation behavior of doing their own work well (Loogma and Nemeržitski, 2014), but also the external behavior of actively contributing to the sports cause (Frost, 2012). So how does moral leadership affect PE teachers’ internal and external innovation behavior? Whether there are differences in the impact of moral leadership on PE teachers’ internal and external innovation behavior is an important question that this study tries to answer. Therefore, based on the classification of PE teachers’ innovation behavior types, it is necessary to explore the mechanism behind it from the perspective of moral leadership. The words and actions of a leader with a particular leadership style will be perceived and assessed by the individual, and will also have a subtle impact on the individual’s work environment, thus influencing the individual’s innovation behavior. Some scholars have proved that moral leadership can improve individual creativity. This impact is not a single path, but is regulated by individual emotional commitment, creative self-efficacy and psychological safety (Iqbal et al., 2022). At the same time, it should be noted that individuals’ identification with leaders directly affects the effectiveness of moral leadership in building individuals’ sense of psychological safety (Tu and Lu, 2013). Therefore, in order to further refine and explore the impact mechanisms of moral leadership and innovation behavior of PE teachers, this study introduces two key variable factors, namely psychological safety and identification with leader, to provide suggestions for enhancing innovation behavior of PE teachers both internally and externally.

## Literature review

### Existing research on moral leadership

#### The concept of moral leadership

Moral leadership refers to two aspects of interpretation of moral people and managers (Treviño et al., 2016), which means that a moral leader is not only a moral person who is altruistic, honest, and trustworthy (Treviño et al., 2016), but also a manager who has the self-discipline to display appropriate conduct, back up his/her words with actions, cares for and respects individuals equally, upholds ethics (Brown and Treviño, 2006), emphasizes mutual communication, and provides instrumental and emotional support (Brown et al., 2005). According to opinions of Brown et al., moral leadership involves high standards of humanistic care, justice, responsibility, and other characteristics that promote followers’ moral standards through mutual communication that passes humility and autonomy between moral leaders and individuals (Brown et al., 2005). Henson proposes that moral leadership exists when a leader maintain a high level of self-perception and behavior that reflects a high level of initiative, and has a positive impact on their followers or society. So far, only Brown and Trevino have articulated a clear definition of moral leadership (Henson, 2015). They believe that moral leadership concerns normative personal behavior and appropriate interpersonal relationships, and usually makes decisions through mutual communication with followers.

#### Trends in moral leadership

For a long time, moral leadership was considered a constituent dimension of other leadership theories (i.e., authentic, democratic, and transformational leadership). Kanungo and Mendonca (1996) first classified moral leadership into three dimensions: a leader’s motivation/intention, influence strategy, and character. Later, Resick et al. (2006) conducted a comparative analysis on the dimensions of moral leadership in different cultures and then adapted the dimensions to inclusive character, integrity, altruism, collective motivation, and encouragement (Resick et al., 2006). Therefore, combining the studies over the years, the dimensions of moral leadership can be summarized into trait dimensions such as motivation, traits, altruism, and behavioral dimensions such as influence strategies, empowerment, and encouragement, among other things.

Existing research suggests that the factors that influence moral leadership are generally personal characteristics, cognitive factors, and situational factors. Based on the work of Brown et al. (2005) and Hoogh and Hartog (2008) found that leaders’ social responsibility, likability, and responsibility promote the formation of moral leadership (Hoogh and Hartog, 2008). In terms of cognition, Mayer et al. (2012) used social cognitive theory to show that ethical identity is an important influencing factor in moral leadership. In terms of situational factors (Mayer et al., 2012), Brown and Treviño (2013) found that role models promote moral leadership in the professional development of leaders (Brown and Treviño, 2013).

Researchers’ understanding of the impact of moral leadership has gradually expanded from followers’ ethical and non-ethical behaviors to their positive behaviors (Hoogh and Hartog, 2008; Tu and Lu, 2013). First, this is generally an antecedent variable that has a direct or indirect positive effect on employees’ attitudes and behaviors in organizations and leads employees to proactively improve the team (Jha and Singh, 2019); furthermore, this is significantly and positively associated with employees’ identification with the leader (Ullah et al., 2022). Moral leadership is also positively associated with employees’ innovation behavior and indirectly stimulates innovation by promoting psychological safety and self-efficacy (Younas et al., 2020). Furthermore, moral leadership can also act as a moderating variable that positively moderates the link between CSR and psychological safety (Kim et al., 2021), while also having a significant moderating effect on the relationship between interactional equity and innovation behavior.

Despite this, few empirical studies have explored the intervening variables between moral leadership and innovation behavior. Moreover, although research on the impact of moral leadership on employee behavior has been studied, its impact on employee innovation behavior is still at an early stage of development compared to other leadership styles. Therefore, based on the existing literature, this study further explores the impact of moral leadership on innovation behavior.

### Existing literature on innovation behavior

#### The concept of innovation behavior

Joseph Schumpeter, an American economist, first proposed the concept of “innovation” in his early book “Theory of Economic Development.” He argued that innovation includes both “invention” and “promotion,” which means that an organization gathers all its internal and external resources to produce new inventions, products, processes, or methods through resource integration (Schumpeter, 1911). Later, as the body of research grew, scholars began to believe that innovation is a process of generating new ideas or products, where innovation behavior is a discontinuous combination of activities including individual innovation behavior, team innovation behavior, and organizational innovation behavior. Among these components, individual innovation behavior is the basis of team and organizational innovation (Ouakouak and Ouedraogo, 2017). Therefore, this paper takes individual innovation behavior as its primary focus.

Research on employees’ innovation behavior began in the 1980s. Since then, scholars have defined “innovation behavior” based on different perspectives, which are shown in Table 1.

**TABLE 1:** Definition of innovation behavior.

Scholar	Definition of innovation behavior
Schumpeter, 1983; Amabile, 1983	The process from the generation of new ideas to the implementation of products, in which employees’ behavior exceeds the expectations of the organization.
West and Farr, 1989	The exploration of new ideas and new processes, and a series of activities to improve organizational efficiency when the innovation results are applied to the organization.
Scott and Bruce, 1994	A multi-stage process of generating innovative ideas, seeking innovative support, and finally turning innovative ideas into reality.
Kleysen and Street, 2001	Individual activities that introduce and practice innovative ideas that are beneficial to organizational innovation.
Tsai and Kao, 2004	The process of generating inspiration in work, developing new products and technologies, implementing the inspiration results of innovation, and finally applying them to form products or services.
Moultrie and Young, 2010	New ideas that are applied to specific work scenarios and produce good results.
Yuan, 2009	A series of complex behavioral activities involving the generation, formation, and implementation of new ideas at work.

As demonstrated, the scholars all agree that the realization of individual innovation behavior needs to go through the stage of generating and forming new ideas, searching for support, and then finally being implemented. Therefore, this paper studies each stage of employees’ innovation behavior from this perspective.

#### Trends in innovation behavior

Although the process theory of individual innovation behavior is recognized by most scholars, there is still no consensus on the division of the dimension of innovation behavior. Some scholars believe that the process includes multiple stages, whereas others believe that the multi-stage activities included in innovation behavior are coherent, related, and can be integrated into one dimension for evaluation and measurement. Therefore, scholars use different application scenarios and classifications to define the different dimensions of innovation behavior. Perhaps the most representative classification is Scott and Bruce’s (1994), which mentioned a three-dimension scale consisting of the generation, promotion, and realization of innovative ideas (Scott and Bruce, 1994). George and Zhou (2001) mentioned a one-dimensional scale referring to supervisors’ subjective evaluation of subordinates’ innovation behavior (George and Zhou, 2001). Kleysen and Street (2001) mentioned a five dimension scale that included searching for opportunities, forming ideas, conducting surveys, seeking support, and applying practices (Kleysen and Street, 2001). Krause (2004) mentioned a two-dimensional scale that centers around generating and executing ideas (Krause, 2004).

The exploration of antecedent factors of innovation behavior is mainly carried out at the individual and organizational levels. The individual level includes personality traits and psychological factors; For example, studies have shown that employees with creative personalities have stronger senses of creativity; Similarly, open personalities are more imaginative in terms of problem discovery and solution, as well as more interested in solving complex or inefficient problems (George and Zhou, 2001). Personality traits determine innovative cognitive styles and the ability to deal with problems by bringing a new perspective (Shalley et al., 2016). In terms of psychological factors, positive emotions help employees generate new ideas in the workplace. High innovative self-efficacy, organizational emotional ability, or psychological capital makes employees behave more innovatively and take a more active role in implementing new ideas (Zhang and Bartol, 2010).

The organizational level includes organizational management and leadership style. Leadership styles are considered to be key to promoting employees’ innovation behaviors. Modest, inclusive, transformative, and authoritative leaders give employees the space and opportunity to develop, while interfering less with their innovative ideas, giving them encouragement and positive feedback, and improving users’ self-evaluation and self-efficacy. At the same time, it provides organizational atmosphere support for the generation and implementation of employees’ innovative ideas (Shao et al., 2022). Furthermore, external learning activities have a positive impact on employees’ innovation behavior (Liu and Gui, 2017). At the same time, the intensity of human resource management has enhanced the positive impact of the organizational atmosphere and the psychological status of innovation behaviors. Having a high-performance work system makes employees more likely to accept the pressure and available support (Ashiru et al., 2022), maximize the generation and implementation of creativity, and enhance innovation performance.

The outcome variables of employee innovation mainly focus on the improvement of organizational efficiency and performance, such as bringing new thinking and methods for the optimization and improvement of operation processes and business efficiency (Ng et al., 2010). Continuous innovation behavior plays a key role in improving the overall performance, competitive ability, and sustainable development of teams and organizations (Liu, 2013).

In recent years, scholars have noticed that the role of leadership style in innovation behavior cannot be ignored (Khan et al., 2021), and, consequently, a series of impact studies have been conducted. Most of these studies have focused on how leadership styles affect the innovation behavior of employees through individual characteristics, which are inextricably linked with cognitive willingness. Therefore, to enrich the research field, it is necessary to consider leaders’ cognition and psychological factors when exploring the influence of leadership style on employees’ innovation behavior.

Furthermore, according to the literature on the dimension division of employee innovation behavior, scholars mostly divide the dimension from the process of behavior development or the evaluation of innovation results. Taking the organization as the boundary to distinguish the internal and external innovation behaviors of employees is helpful to further explore the differential influence mechanism of moral leadership on individual innovation behaviors, and build a targeted incentive mechanism for employees’ innovation behaviors.

### Existing literature on psychological safety

#### The concept of psychological safety

In this context, psychological safety is defined as employees being able to be themselves without the subjective perception that their self-image, status, or career will be adversely affected (Kahn, 1990). This concept is extended to the team level, and defined as the shared belief that team members can safely take interpersonal risks (Edmondson, 2016). In the workplace, psychological safety represents an environmental state that provides enough certainty and predictability for employees to exert their creativity (Gong et al., 2010). Psychological safety is an important index to measure the degree of individuals’ ability to adapt.

#### Trends in psychological safety

Regarding the dimensional division of psychological safety, Edmondson (1999) considered psychological safety as one dimension scale measured by psychological safety from a team’s perspective (Edmondson, 1999), while Tynan (2005) extended this understanding by dividing individual team members’ psychological safety into two dimensions: self-psychological safety and others’ psychological safety (Tynan, 2005). The former refers to the degree of safety an individual feels when a specific other person is involved, whereas the latter refers to an individual’s perception of the degree of psychological safety of others. Thus, Edmondson (1999) focused on the psychological safety of the team between the individual and the team (Edmondson, 1999), and Tynan (2005) focused on the psychological safety of the self and the psychological safety of others, which more comprehensively accounts for the relationship between an individual, his/her colleagues, and his/her supervisors.

Existing studies have examined the antecedents of psychological safety, such as transformational leadership, servant leadership, and moral leadership. Shin and Zhou (2003) stated that transformational and ethical leaders are able to create a situational climate of psychological safety within teams (Shin and Zhou, 2003; Walumbwa and Schaubroeck, 2009). In terms of outcome variables, psychological safety is an important factor that influences employees’ engagement behaviors, individual/team learning behaviors, innovation behaviors like new ideas (Carmeli et al., 2014), constructive behaviors (Carmeli et al., 2010; Edmondson, 2016), and knowledge sharing (Siemsen et al., 2009). Moreover, psychological safety acts as a mediating variable between management style and creativity (Jiang and Gu, 2016); corporate social responsibility and employee creativity (Kim et al., 2021); and ethical leadership and innovation behavior (Jin et al., 2022). Current research has focused on the effects of psychological safety on employees’ and teams’ learning, innovation, and performance; However, it has not sufficiently explored the effects of other factors at the individual level. Most existing studies have been conducted with psychological safety as a mediating or moderating variable, despite there being no systematic theory to draw on.

### Existing literature on identification with leaders

#### The concept of identification with a leader

Leadership identity is constructed based on the pride that is generated by employees that admire their leaders’ attitudes and behaviors, which leads to the formation of a sense of identity. Becker (1992) and Pratt (1998) divided subordinates’ identification with their leaders into two types: One is for employees to realize that their values are similar to those of their leaders, and the other is for employees to change their values per their leaders (Becker, 1992; Pratt, 1998). Knippenberg and Hogg (2003), on the other hand, saw identification with leader as the process by which subordinates form a perception of themselves, a process that depends on the role of the leader in their relationship (Knippenberg and Hogg (2003)). Identification with a leader is the tendency of individuals to identify with, and be willing to accept, a leader’s norms or to be protective of the leader (Brown et al., 2005). Currently, Pratt (1998), who holds that employees’ identification with their leaders is equivalent to the process by which the beliefs that employees have about their leaders are transformed into self-definitions, is widely used (Pratt, 1998).

#### Trends of identification with leader

Current studies consider identification with leaders to have a one-dimensional structure, without making a multidimensional delineation. In view of social identity theory, identification with a leader is understood as leadership styles affecting employees’ cognition, attitudes, and behavior (Yang et al., 2020). Among the different types, moral leadership treats employees fairly and considers their needs; This is felt by employees and consequently stimulates their sense of identity in alignment with their leaders. Some scholars consider there to be a significant positive correlation between moral leadership and identification with leaders (Lee, 2016). In terms of outcome variables, identification with a leader impacts employees’ work attitude, behavior, and performance (Lührmann and Eberl, 2016). Some scholars have considered it a mediating variable between inclusive leadership and employee voice or prosocial behavior (Khattak et al., 2022), or between moral leadership and inhibitory feedback. Furthermore, existing studies take identification with a leader as a moderating variable between charismatic leadership and innovation performance of employees (Zhao et al., 2021). Current research has mainly used identification with leaders as a mediating variable, while the amount of literature on identification with leaders as a moderating variable is small and needs to be expanded.

In summary, moral leadership affects identification with leaders and psychological safety. Over the years, scholars have constantly proposed and enriched the dimensional division of moral leadership, which can be summarized in terms of two perspectives: traits and behaviors. This is also an important aspect of individuals’ cognition and judgment on leadership, and an important basis for employees to maintain a positive attitude in the organization (Karim and Nadeem, 2019). Identification with leaders and psychological safety mediate the relationship between leadership style and innovation behavior. Identification with leader refers to individuals’ cognition similar to the leader’s value orientation or their willingness to follow the leader’s value orientation, which affects employees’ self cognition, attitude and behavior (Mitonga-Monga and Cilliers, 2016). Psychological safety reflects the status of individuals’ working environment to some extent, which needs to be guided and maintained to ensure individuals’ innovation stability (Walumbwa and Schaubroeck, 2009). Looking at the above, both the individual level and the organizational level will affect the realization of individual innovation behavior. Among them, the leadership style plays an important role in influencing the relevant dimensions. A leadership style not only affects the management atmosphere and corporate culture at the organizational level (Xie et al., 2018), but also imperceptibly guides and affects employees’ value identification from a cognitive perspective (Frolova and Mahmood, 2019). Currently, although some scholars have discussed the impact of moral leadership on employee behavior, research in this field is still at an early stage compared to other research on leadership styles, and empirical studies are relatively scarce. Most of the research on the influence of leadership style on individual innovation behavior has focused on the influence of leadership style on the creative characteristics of individuals, with relatively monolithic research perspective and impact path. It will further enrich the scope of exploration in this field by increasing the consideration of individual identification with a leader and psychological safety in an organization.

## Hypotheses development

### Theoretical basis

#### Social responsibility theory

Social responsibility theory refers to the responsibility and obligation of enterprises to pay attention to the rights and interests of all stakeholders. The beneficiaries scope of responsibility includes employees, managers, partners, or competitors. This theory has been widely used to study the relationship between organization and individual, individual identification (George et al., 2020), and individual innovation behavior (Li et al., 2020). In this paper, we focus on the effect of moral leadership on PE innovation behavior. Moral leadership is the representative of the transmission of organizational values and social responsibility, and PE innovation behavior is generated by groups in PE education. This theory is consistent with the driving goal of the impact of moral leadership on individual innovation behavior in this study.

Some studies have shown that the organization’ social responsibility for employees’ benefit enhances the individual’s organizational emotional commitment and drives them to produce behavior that is conducive to organizational development and innovation (Bouraoui et al., 2019). Based on this theoretical framework, individuals perceive more organizational support and form a strong internal innovation motivation (Khan et al., 2021), at the same time, under the guidance of the social responsibility theory, it is easier to form a flexible and inclusive innovation atmosphere within the organization, providing individuals with a wider space for self-expression. Under this organizational atmosphere, employees have less pressure and show more active innovative thinking and behavior output. That is, the higher the corporate social responsibility of an organization, the higher the leadership’s moral identity and organizational trust, and the stronger the individual’s innovation ability (Hur et al., 2018).

#### Social cognitive theory

Social cognitive theory refers to the individual’s initiative behavior driven by internal cognition, which is an integration of personality theory and cognitive theory (Bandura, 1989). This theory is widely used to explain career activities, including self-efficacy, workplace attitude, and intention to leave or stay (Zikic and Saks, 2009), explore the psychological mechanism of individual initiative, and its impact on behavior, balance the relationship between self and others (Bandura, 1989).

The theory emphasizes the relationship between environment, individual and behavior. In particular, personal attitudes and psychological characteristics will be activated by some factor in the external environment, such as leadership (Judge and Zapata, 2015), thus creating an internal driving force for personal behavior. Studies have shown that individuals’ identification with moral leadership directly affect their loyalty and sense of mission to the organization. When individuals more cognitively identify with their leaders, they show stronger team cohesion, maximize the communication with moral leaders (Nejati et al., 2021), improve their self creativity effectiveness and sense of contribution, make them feel more positive about their work and create more innovative ideas in the organization (Griffin et al., 2010).

#### Social learning theory

Social learning theory holds that people learn most behavioral patterns by observing and imitating the behavior and behavior outcomes of others. This theory attaches importance to the role of role models, and believes that what kind of behavior an individual obtains in the organization and the quality of behavior performance depend on the role of role models (Ahn et al., 2020). As far as enterprise management is concerned, the leader’s behavioral patterns and handling style determine whether his followers behave as expected by the organization, whether they have a high level of enthusiasm for their work and a sense of mission to creatively solve problems. This theory is usually used to explore the impact on individual behavior in a specific environment and individual imitation behavior, which can help understand the psychological reaction mechanism of followers (Le and Hancer, 2021), and is consistent with the process research on the impact of the relationship between leadership and employee behavior (Yarberry and Sims, 2021).

This theory emphasizes that individual behavior is the result of the interaction between self cognition and external environment, and a series of individual behaviors will be gradually formed through learning and imitation (Wu et al., 2020). Leaders’ moral standards and behaviors will affect employees’ psychology and behaviors. Employees under moral leadership show more organizational citizenship behaviors (Hoogh and Hartog, 2008), form a strong sense of responsibility and follow the leader’s willingness to contribute to the organization and society, which is conducive to innovation behavior by individuals both inside and outside the organization.

#### Social exchange theory

The theory of social exchange holds that individual experiences balance their rewards and costs in social interaction through self-regulation (Cook and Rice, 2001). This theory is often used to analyze the reciprocal exchange ability between groups (McLeod et al., 2021), and is also widely used to explain workplace relations and individual behavior (Cropanzano and Mitchell, 2016), such as the research on the impact of moral leadership and employee loyalty. When individuals obtain any beneficial reward in the organization, including identification, material acquisition, status and reputation, it will also affect their behavior that benefits the organization (Fan et al., 2021). The application of this theory provides a new interpretation of the psychological mechanisms that probe the relationship between moral leadership and innovation behavior.

This theory emphasizes the feedback psychology and behavior of the beneficiary group. When employees obtain economic and social emotional resources inside and outside the organization, their sense of responsibility in repaying the organization and society will be stronger (Cropanzano and Mitchell, 2016). That is, when employees perceive the organizational support and care conveyed by moral leaders, they will adjust their social exchange ideology, improve their own engagement and sense of mission, and generate a strong sense of self-efficacy, thus stimulating the innovation enthusiasm of continuous breakthrough, and generating more active innovation behavior to feedback and develop the organization.

The four aforementioned theories complement each other in terms of the relationship between the explanatory variables, although each emphasizes a different stage of the process. For example, social responsibility theory emphasizes the role of a moral atmosphere or moral leadership for the sake of individual innovation behavior. In contrast, social learning theory emphasizes the interaction between external stimuli (such as leadership) and individual behavior. Social cognition theory embodies the intermediate state of this interaction process and complements the influence process of individual cognition on their behavior. The three theories comprehensively link the logical chain and effectively explain the process mechanism of leadership styles based on individual leadership cognition and psychological safety in their innovation behavior. Finally, social exchange theory further complements the psychological adjustment process of employees’ innovation behavior, refines the realization mechanism of cognitive and behavioral processes, and makes the hypothesis deduction logic of this study more rigorous and coherent.

### Hypotheses development

#### The relationship between moral leadership and innovation behavior

According to social learning theory, the role of followers is regulating their own behavior based on their leader’s behavior by observing the leader’s cognition and characteristics. Moral leaders form a good role model in the organization through their motivational language, values expression, etc., provide an example for followers to learn, create an innovation atmosphere, and influence individuals’ values and behavior through moral communication and behavior guidance, thus promoting individuals to achieve unique innovative contributions beyond their roles (Abu Bakar and Connaughton, 2022). Employees under moral leadership tend to have more altruistic and value creating behaviors, such as a sense of responsibility for the society and groups outside the organization, which encourages employees to find problems and participate in solving problems in other environments outside the society or the organization. Moral leadership affects individuals’ cognition and belief through its value influence, and then affects their motivation, attitude and behavior (Bal et al., 2014). Previous studies have shown that moral leadership is a major source of the imagination for individuals and organizations. It promotes the interconnection between individuals and organizations and is the main driving force for continuous innovation at the individual and organizational levels. According to the theory of social exchange, organizational care delivery and affirmative support represented by leadership performance will, to a large extent, stimulate employees’ organizational feedback psychology and willingness to contribute to themselves, and improve the overall creativity of the team (Wang et al., 2021). The interaction between employees and moral leaders can make employees have a strong sense of trust in the organization. As an important channel for the organization to convey employees’ feelings, moral leaders give employees sufficient emotional support and innovation tolerance, enhance employees’ innovation vitality and reward motivation (Chughtai, 2016), and show more frequent implementation of innovative ideas and behaviors (Chen et al., 2022; Saeed et al., 2022). Therefore, moral leaders encourage individuals to obtain opportunities and solutions for self-development (Tang, 2017). According to the guidance of the two theories, it can be found that moral leadership in the organization will drive employees to find problems inside and outside the organization, generate creative ideas and innovation behaviors, and finally solve problems (Cohen-Meitar et al., 2009). Based on this, the following hypothesis is proposed:

H1: Moral leadership enhances innovation behavior.

H1a: Moral leadership enhances internal innovation behavior.

H1b: Moral leadership enhances external innovation behavior.

#### The relationship between moral leadership and psychological safety

According to social exchange theory, fair, caring, and sincere communication by leaders can create trust (He et al., 2020). Moral leaders can build mutual respect and trust with individuals by prioritizing individuals’ needs, establishing open communication (Resick et al., 2013), treating individuals fairly (Mo et al., 2019), and respecting individual interests to enhance psychological safety (Newman et al., 2014). Moral leaders’ integrity, honesty, and frankness are key foundations of employee trust. Employees gain job security when they trust that their leaders will fairly evaluate their efforts and reward them as they deserve (Schwepker and Dimitriou, 2021), which also affects psychological security (Walumbwa and Schaubroeck, 2009). Additionally, according to social learning theory, moral leaders impact employees’ psychological safety by holding up moral standards and creating credibility; In turn, subordinates identify with the values of their leaders, and thus they imitate their behavior (Bandura, 1989), and conform to their ethical standards. Individuals experience a high level of psychological safety when they develop a mutually supportive and trusting relationships with their leaders (Kahn, 1990; Ahmad and Umrani, 2019). By reducing the chances of negative consequences on an employee, moral leadership enhances psychological safety (Younas et al., 2020). Moral leaders eliminate individuals’ worries (Chen et al., 2022), thus playing a key role in their psychological safety (Liu et al., 2016). Based on this, the following hypothesis is proposed:

H2: Moral leadership enhances psychological safety.

#### The relationship between psychological safety and innovation behavior

Based on the social responsibility theory, an organization has the obligation to fulfill social responsibility to its stakeholders, especially the hired individuals, while realizing its own sustainable operation and development. Individuals are valuable assets and wealth of an organization. The organization should provide a professional environment conducive to personal development and a humanistic environment more caring for individual welfare (Glavas, 2016), ensure the psychological safety of individuals in the organization, enhance the staff’s stability and sense of mission, and provide sufficient and safe space for individuals to carry out innovative activities (Zhong et al., 2022). As representatives of an organization, leaders have the obligation to create a management atmosphere that cares for and supports individuals, and stimulate individuals’ sense of social responsibility, which is the key to realizing individuals’ sustainable innovation behavior (Broch et al., 2020), which helps to build a strong sense of trust between employees and leaders, establish employees’ sense of belonging and loyalty, improve employees’ sense of psychological safety, positively affect employees’ generation and free expression of non-traditional views in work, and generate innovation behaviors. A positive psychological safety guarantee may be a new way to encourage employees to better understand the close relationship between them, the organization and the wider society, find problems, and innovate and implement boldly within the organization. Therefore, the following hypotheses are proposed:

H3: Psychological safety enhances innovation behavior.

H3a: Psychological safety enhances internal innovation behavior.

H3b: Psychological safety enhances external innovation behavior.

#### The mediating role of psychological safety

In social responsibility theory, moral leadership helps build mutual trust and respect, while also providing individuals with flexible time and space and creating a supportive organizational culture. Moral leaders with qualities such as integrity and responsibility can create a fair and comfortable organizational climate for individuals (Byun et al., 2018). According to social cognitive theory, leaders’ support, trust, and respect make individuals feel that they can rely on moral leaders and strengthen their psychological safety (Edmondson, 2016; Hu et al., 2018). This moral environment ensures that individuals are tolerated in case of any mistakes or misunderstandings, so that individuals have an increased sense of psychological safety, and individuals feel comfortable and easily come up with new ideas (Walumbwa and Schaubroeck, 2009). In addition, moral leaders promote mutually open communication, listen sincerely to their followers, encourage them to voice their concerns and opinions (Hu et al., 2018), and create a sense of psychological safety for individuals, thus inspiring them to come up with novel ideas (Martins and Terblanche, 2003; Tu and Lu, 2013). Therefore, moral leadership will not only have a direct impact on individuals’ psychological safety and innovation behavior, but may also indirectly influence the hired individual’s innovation behavior through the mediating role of psychological safety. Therefore, the following hypotheses are proposed:

H4: When moral leadership guides employees’ psychological safety, it increases innovation behavior.

H4a: When moral leadership guides employees’ psychological safety, it increases internal innovation behavior.

H4b: When moral leadership guides employees’ psychological safety, it increases external innovation behavior.

#### The moderating role of identification with a leader

Employees’ identification with their leaders is a powerful way of influencing individuals (Avolio et al., 2004), and an important potential variable in the effectiveness of leadership management (Conger and Kanungo, 1994). According to social identity theory and social learning theory, individuals have identification with leader when their basic needs (e.g., a sense of belonging) are met (Zhu et al., 2015). Individual’s identification with leaders will mean that employees take their leader’s advice as a reference, which motivates them to realize similar values and change their existing cognitive concepts (Tse and Chiu, 2014; Zhu et al., 2015). At the same time, they will behave appropriately and per the organization’s goal, rather than challenging the leader (Miao et al., 2013). The stronger the employee’s identification with the leader, the weaker the negative perception and feeling of moral leadership (Giessner et al., 2015). Therefore, based on the above two theories, high leadership recognition will encourage employees to adopt the good moral character and behavior of their leaders, work following their values, and not worry about being punished, thus enhancing their psychological security. On the contrary, employees with low recognition of the leader are unlikely to agree with the leader’s values and may be less likely to try and emulate or acquire the leader’s qualities. Furthermore, when the identification with a leader is low, the direct and positive influence of the leadership on individuals’ feeling will be limited (Peng and Rode, 2010). Therefore, low leader recognition will hinder the influence of moral leadership on employees, thus increasing the uncertainty in work and weakening the psychological safety that results from moral leadership. Therefore, the following hypothesis is proposed:

H5: Identification with a leader positively influences the impact of moral leadership on psychological safety.

**FIGURE 1:**
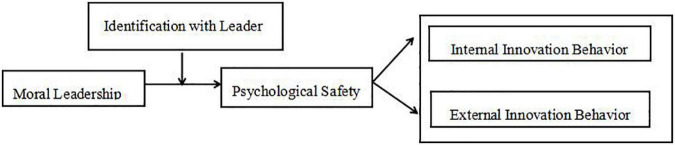
Research framework.

Based on the above, the research model of this study can be visualized as follows (see Figure 1):

## Materials and methods

### Sample and process

In order to better address the research questions and to fully reflect the process of influence of moral leadership on the innovation behaviors of PE teachers, we ultimately selected Chinese teachers as the study sample for the following reasons. The first reason is the uniqueness of the study sample. In educational settings, the exploration of the relationship between individual’s innovation behaviors and achievement performance has been conducted mainly in primary and secondary schools in western countries, with a special focus on teachers’ innovation behaviors in teaching and there is a lack of in-depth research on Chinese PE teachers’ innovation behaviors (Gao and Han, 2022). Second, regarding the availability and authenticity of the data, China was the most convenient country for our research team to obtain the data, as we are able to interface with the Chinese education authorities to obtain the data we need, which enhanced the authority of the data collected in this study. Finally, China is an early promoter in sports innovation strategies, and PE teachers are practitioners at the forefront implementing new strategies, and their innovation behaviors are critical to the sustainable development of school profession, help the public to form a lifelong exercise habit and offer inspiration for the sports industry, so this research selects Chinese PE teachers as samples to observe their innovation behavior. Only people are in top physical, mental and psychological shape, laying the foundation for China’s scientific and technological advancement.

Therefore, this study investigated PE teachers from different regions, schools, and positions in Chinese provinces (namely Fujian, Guangdong, Henan, Jilin, Beijing, Hainan, Anhui, Jiangxi, Liaoning, Hunan, Zhejiang, Jiangsu, Shandong, Shanxi, Shanghai, Guizhou, Hebei, and Heilongjiang Province). To this end, a questionnaire was designed based on an initial interview that was conducted with PE teachers, and a small-scale pre-survey that ensured the accuracy of the questionnaire items. A total of 327 questionnaires were distributed, and 287 valid responses were collected. The questionnaire was divided into six parts; The first part collected respondents’ demographic data (i.e., gender, age, and educational level), and the other five parts focused on moral leadership, psychological safety, identification with leaders, and two kinds of innovation behaviors (internal innovation behavior and external innovation behavior). Based on the collected data, this study analyzed and clarified the relationship between PE teachers’ moral leadership, psychological safety, identification with leaders, and two kinds of innovation behaviors. The study used SPSS to conduct the statistical analysis of the data and to analyze the mechanism of moral leadership on PE teachers’ innovation behavior from an empirical perspective.

The responses were measured on a 5-point Likert scale (5 points for “very much agree”; 4 points for “relatively agree”; 3 points for “uncertain”; 2 points for “relatively disagree”; and 1 point for “very much disagree”).

This study chose respondents of different genders, ages, and education levels. The final sample contained more males than females, but the gap was not large. Additionally, respondents were characterized into the following age groups: 45–54 years old (3.83%), 35–44 years old (16.03%), 25–34 years old (39.02%), 24 years old, and below (3.48%). In terms of education level, 2.79% had a bachelor’s degree, 70.03% had a master’s degree, 24.39% had a doctor’s degree, and 2.79% fell into none of the categories. The education level distribution was symmetrical and covered all types, which improved the accuracy of data collection. Thus, the data collection was deemed reliable and comprehensive.

### Data measurement

#### Scale of moral leadership

To measure ethical leadership, Khuntia and Suar (2004) developed a 22-item scale based on the dimensions of empowerment, motivation, and character (Khuntia and Suar, 2004). The scale includes “willingness to coach and guide hard-working employees” and “rewards and punishments for subordinate behavior.” Among the existing studies, Brown et al. (2005) have the most widely used definition and measurement of ethical leadership. According to their work, moral leadership refers to the ethical behaviors in personal and interpersonal relationships, and a decision making process that includes two-way communication, to encourage followers to have similar moral behaviors (Brown et al., 2005). Brown et al. (2005) developed a 10-item scale that included items such as “making fair and just decisions” and “modeling how to handle things ethically and correctly.” Huang and Paterson (2017) developed a 10-item scale based on Brown et al.’s (2005) previous work and measured its reliability in two samples that had Cronbach’s α of 0.93 and 0.95 (Huang and Paterson, 2017). The representative question was: “Success is not only measured by outcomes, but also by the way in which the outcomes are achieved.” We selected widely used indicators, integrated the above representative scales, and made final measurement indicators that included valuing the rationality of process and method, communicating with employees or giving them reasonable advice, emphasizing fair reward and punishment, and balancing the interests of all parties, clarifying the ethical principle and possible consequences of unethical behavior, respecting subordinates, and giving guidance, support and help to subordinates.

#### Scale of psychological safety

The most widely used measurement of psychological safety is Edmondson’s Psychological Safety Scale, which includes seven items that were developed to measure the psychological safety of teams (Edmondson, 2016). This scale has been directly quoted or revised by subsequent researchers and is represented by the statement: “I am able to bring up problems and tough issues in this organization.” To measure individual psychological safety, Carmeli et al. (2010) modified the Team Psychological Safety Scale to create the Individual Psychological Safety Scale (Carmeli et al., 2010), which consists of five items including “It is easy for me to ask other members of this organization for help” and “’It is safe to take a risk in this organization.” Liang et al. (2012) focused on personal perceptions of psychological safety (Liang et al., 2012), and created five items to measure this based on previous studies (Brown and Leigh, 1996), including “Nobody in my unit will pick on me, even if I have different opinions.” In this study, we selected widely used indicators, integrated the above representative scales, and made final measurement indicators that included being accustomed to seeking help from leaders or other members of the unit, feeling comfortable with the common beliefs and high cohesion between team members, and having strong certainty, predictability, and confidence in the future work.

#### Scale of identification with a leader

In regards to identification with leaders scales, Mael and Ashforth (1992) developed a six-item scale to measure identification with leaders (Mael and Ashforth, 1992), which is represented by questions that take the subject “We” rather than “they” when speaking about leaders. Furthermore, Shamir et al. (1998) developed a seven-item scale to measure identification with leaders, which includes statements such as “My values are similar to his/her values” and “He/she is a model for me to follow”(Shamir et al., 1998). Kark et al. (2003) developed identification with leaders scale based on Shamir et al.’s (1998) previous research using a sample of bank employees (Kark et al., 2003), whose representative item was “I see my leader’s success as my own.” Here, the internal consistency reliability of this scale was 0.96. We selected widely used indicators, integrated the above representative scales, and made final measurement indicators that include habitually using the term “my leader” instead of “him/her,” feeling happy when someone praises my leader, the leader is important to me and is a role model, etc.

#### Scale of internal innovation behavior

As for the scale of internal innovation behavior, Scott and Bruce (1994) measured employees’ innovation behavior in an organization from the perspectives of identifying a problem, generating a conception, seeking innovation support, and implementing an innovation plan (Scott and Bruce, 1994). The scale includes six items. The Cronbach α is 0.89. Similarly, another scale was created by George and Zhou (2001), which measures the performance of employees’ innovation behaviors from the perspective of supervisors and better reflects the process of employees’ innovation (George and Zhou, 2001). Through the comprehensive arrangement of both aforementioned scales on internal innovation behavior, a three-dimensional scale for employees was formed, which includes aspects such as understanding and finding problems; searching or adopting new methods and processes; and dialectically thinking about problems.

#### Scale of external innovation behavior

As external innovation behavior involves sharing and patching internal and external resources, as well as the search and internalization of external innovation knowledge, both the organization and the social benefit. Therefore, to measure the external innovation behavior dimension, we refer to the open innovation scale of Hung and Chou (2013), whose representative topics are “enterprises often acquire and apply external technical knowledge, and enterprises actively promote internal knowledge to the external market” (Hung and Chou, 2013). In Dong and Netten (2017)’s external innovation knowledge search width and depth scale and Laursen and Salter’s (2006) knowledge acquisition channels and degrees scale, the representative topics are “enterprises actively seek new technology fields and understand external problems to be solved,” “enterprises search for and acquire innovation knowledge through suppliers, customers, competitors, higher education institutions and other channels” (Laursen and Salter, 2006). Finally, Scott and Bruce’s (1994) employee innovation behavior scale includes “seeking support and assistance” and “a path to realizing innovative ideas.” The combination of scales in a five-dimensional external innovation behavior scale of individuals (Scott and Bruce, 1994), including using experience to solve social or external problems, understanding and discovering the problems to be solved urgently by society and external organizations, supporting creativity and solving problems through win-win cooperation of external resources.

### Methodology

First, this research has clear questions and research hypotheses at the beginning of the study, quantitative research is more in line with the research process, which derives the intrinsic relationships among the variables, verifies the research hypotheses one by one and helps to explore the truthfulness and rationality of the theoretical hypotheses proposed in this study. Moreover, in contrast to the qualitative analysis methods, hierarchical multivariate regression analysis, this quantitative analysis method, has the advantage of empirical evidence, clarity and objectivity, and the results are more intuitive. Secondly, this research uses data collection method of questionnaire survey to promote the immediacy and reliability of data collection in moral leadership (ML), psychology safety (SS), identification with leaders (IL), internal innovation behavior (IB), and external innovation behavior (EB). Finally, this research uses the software SPSS 25.0 to process the collected data, analyze the overall situation of ML, SS, IL, IB, and EB and the correlations among these variables.

### Data analysis

As illustrated in Table 2, ML is significantly positively correlated with SS (*p* < 0.01) and positively correlated with IB, and EB (*p* < 0.01). Identification with leaders (IL) is significantly positively correlated with SS. The correlation analysis lays the foundation for further research on the causal relationship between variables.

**TABLE 2:** Means, standard deviations, and correlation coefficient analysis of the variables.

	*M*	SD	1	2	3	4	5	6	7	8
1. Gender	1.362	0.482	1							
2. Age	2.777	0.892	0.132*	1						
3. Education	3.272	0.557	–0.082	–0.068	1					
4. IB	4.347	0.602	–0.070	–0.035	0.003	1				
5. EB	4.010	0.690	–0.050	–0.002	–0.023	0.570**	1			
6. ML	3.843	0.803	–0.042	0.005	–0.106	0.447**	0.448**	1		
7.SS	3.779	0.713	–0.062	0.047	–0.071	0.443**	0.472**	0.589**	1	
8. IL	3.780	0.802	0.035	0.045	–0.064	0.302**	0.328**	0.646**	0.527**	1

*p < 0.05, **p < 0.01.

#### Reliability analysis

SPSS 25.0 was used to test the reliability of the questionnaire data. Cronbach’s α values for IB, EB, ML, SS, and IL were 0.813, 0.879, 0.869, 0.784, and 0.815, respectively (see Table 3); The α values for each variable were all greater than 0.75, thereby confirming the reliability of the data.

**TABLE 3:** Reliability statistics for each variable.

Factor	Cronbach’s a coefficient	AVE	CR
IB	0.813	0.6	0.8
EB	0.879	0.7	0.9
ML	0.869	0.6	0.9
SS	0.784	0.6	0.8
IL	0.815	0.6	0.8

#### Validity test

For validity testing, the Kaiser-Meyer-Olkin (KMO) and Bartley spherical tests were used (see Table 4). The value was 0.897, and the p value was less than 0.01; Thereafter, confirmatory factor analysis was conducted to ensure that the scale data were suitable for factor analysis. The results in the CFA table clearly demonstrate that all variables have the following characteristics: the value of χ^2^/df is less then 3, RMSEA is less then 0.08, RMR is less then 0.05, the value of CFI and IFI are both higher than 0.9, SRMR is less then 0.1 (see Table 5). Overall, the research questionnaire content and study model is well constructed.

**TABLE 4:** KMO and Bartlett test.

Kaiser-Meyer-Olkin	0.897
Bartlett test	2837.837
df	136
Sig.	0

**TABLE 5:** Confirmatory factor analysis.

Common indicators	χ^2^/df	RMSEA	RMR	CFI	IFI	SRMR
IB	<3	<0.08	<0.05	>0.9	>0.9	<0.1
EB	<3	<0.08	<0.05	>0.9	>0.9	<0.1
ML	<3	<0.08	<0.05	>0.9	>0.9	<0.1
SS	<3	<0.08	<0.05	>0.9	>0.9	<0.1
IL	<3	<0.08	<0.05	>0.9	>0.9	<0.1

#### Common method deviation test

The Harman single factor test was used to determine whether there was a common method deviation in the collected data. All items on the six-variable measurement scale were included in the exploratory factor analysis. The results showed that there were five factors (i.e., more than one) with a characteristic root greater than one, and the maximum factor variance interpretation rate was 17.72 (less than 40%). Hence, there was no serious common method deviation in this study (see Table 6).

**TABLE 6:** Total variance analysis.

Component	Initial eigenvalues			Extraction sums of squared loadings		
	Total	% of variance	Cumulative%	Total	% of variance	Cumulative%
1	7.292	42.893	42.893	4.380	25.764	25.764
2	2.098	12.341	55.233	2.535	14.911	40.674
3	1.113	6.545	61.778	2.413	14.195	54.869
4	1.037	6.097	67.875	2.211	13.006	67.875
5	0.959	5.639	73.514			
.	.	.	.	.	.	.
17	0.128	0.751	100.000			

## Results

### The mediating role of psychological safety

SPSS software was used to analyze the mediating effect of PE teachers’ SS between ML and IB, and between ML and EB. The results showed that ML had a significant positive impact on IB (β = 0.338, *p* < 0.01) (see Table 7), a significant positive impact on EB (β = 0.386, *p* < 0.01) (see Table 7), and a significant positive impact on SS (β = 0.519, *p* < 0.01). ML creates a harmonious, free, and innovative atmosphere of an organization for individuals (Xie et al., 2018), guarantees individuals to freely carry out IB and EB, and also provides individuals with strong organizational guarantees to maintain individuals’ SS in the organization (Hu et al., 2018). Thus, H1a, H1b, and H2 were supported.

**TABLE 7:** Mediating effects of IB (n = 287).

	IB			SS			IB		
	*B*	*t*	*p*	*B*	*t*	*p*	*B*	*t*	*p*
Constant	3.021**	10.104	0.000	1.806**	5.643	0.000	2.598**	8.493	0.000
Gender	–0.054	–0.808	0.420	–0.066	–0.914	0.361	–0.039	–0.596	0.552
Age	–0.020	–0.543	0.587	0.039	1.009	0.314	–0.029	–0.820	0.413
Education	0.048	0.831	0.407	–0.013	–0.206	0.837	0.051	0.910	0.364
ML	0.338**	8.426	0.000	0.519**	12.112	0.000	0.216**	4.506	0.000
SS							0.234**	4.343	0.000
*R* ^2^	0.206			0.350			0.256		
*F*	*F* (4,282) = 18.245, *p* = 0.000			*F* (4,282) = 38.014, *p* = 0.000			*F* (5,281) = 19.293, *p* = 0.000		

*p < 0.05, **p < 0.01.

After adding the intermediary variable of SS, the positive regression coefficient of SS on IB was significant, and the impact of ML on IB and EB was still significant (β = 0.216, *p* < 0.01; β = 0.226, *p* < 0.01) (see Tables 7, 8). Therefore, SS has a partial mediating effect between ML and two kinds of innovation behaviors. SS of individual provides positive psychological protection for their innovation behavior (Lee, 2016). Moral leaders can encourage individuals to actively participate in internal and external innovation activities by maintaining the SS of individuals. Thus, H3a, H3b, H4a, and H4b were supported.

**TABLE 8:** Mediating effects of EB (*n* = 287).

	EB			SS			EB		
	*B*	*t*	*p*	*B*	*t*	*p*	*B*	*t*	*p*
Constant	2.490**	7.251	0.000	1.806**	5.643	0.000	1.932**	5.559	0.000
Gender	–0.042	–0.548	0.584	–0.066	–0.914	0.361	–0.022	–0.297	0.767
Age	0.001	0.020	0.984	0.039	1.009	0.314	–0.011	–0.281	0.779
Education	0.028	0.420	0.675	–0.013	–0.206	0.837	0.032	0.499	0.618
ML	0.386**	8.396	0.000	0.519**	12.112	0.000	0.226**	4.151	0.000
SS							0.309**	5.037	0.000
*R* ^2^	0.203			0.350			0.269		
*F*	*F* (4,282) = 17.909, *p* = 0.000			*F* (4,282) = 38.014, *p* = 0.000			*F* (5,281) = 20.640, *p* = 0.000		

*p < 0.05, **p < 0.01.

Based on a comparative analysis of the mediation effects of IB and EB, this study found that the influencing mechanism is more explanatory and effective in PE teachers’ external innovation process. The regression coefficient of SS for EB (shown in Table 8) is larger than that of IB (shown in Table 7). Moreover, the p value of the mediating effect is more significant in the influencing path “ML ≥ SS ≥ EB” (see Table 9), in which the effect proportion ratio is higher. ML will drive and strengthen employees’ perception of psychological capital and social capital; Psychological capital and social capital perception are important factors to buffer employees’ psychological insecurity and enhance their sense of belonging and willingness to contribute (Probst et al., 2017), affect employees’ social innovation tendency (Ullah et al., 2022), and enhance the IB of employees outside the role (Pasricha and Rao, 2018). It can be seen that the impact of ML on individuals’ EB through SS is significantly better than the impact of ML on individuals’ IB, and SS plays a highly important role in ensuring individuals’ innovation behavior beyond their roles and responsibilities (Bammens, 2016). That also means that ML can stimulate employees’ EB by strengthening SS.

**TABLE 9:** Summary of the mediating effect test results.

Influencing path	c Total effect	a	b	a × b					c’	Test result	Effect proportion ratio
				Mediating effect	Boot SE	z	p	95% BootCI	Direct effect		
ML → SS → IB	0.338**	0.519**	0.234**	0.122	0.043	2.859	0.004	0.083–0.249	0.216**	Partial	36.053% (a × b/c)
ML → SS → EB	0.386**	0.519**	0.309**	0.160	0.044	3.673	0.000	0.105–0.277	0.226**	Partial	41.518% (a × b/c)

*p < 0.05, **p < 0.01.

### Moderating effect of identification with a leader

From the perspective of ML, by taking SS as the outcome variable and adding IL, as well as the interaction between IL and ML, we can see that the main effect was not changed after adding the interaction item. However, after adding all variables, the analysis results showed that the coefficient of IL and its interaction was significant (see Table 10). That is, IL played a moderating role in the relationship between ML and SS. The stronger the IL of individuals, the stronger the positive impact of ML on SS (Giessner et al., 2015). An atmosphere of high IL is more likely to create a safe environment consisting of trust and mutual respect, where individuals are free to present their opinions, needs, feelings, ideas, innovative strategies, etc. IL of Individuals affects the transformation effect of ML on the construction of individuals’ SS (Tu et al., 2019), and determines the strength of individuals’ SS under the impact of ML. Thus, H5 was confirmed.

**TABLE 10:** Moderating effects of identification with leaders.

	M1			M2			M3		
	*B*	*t*	*p*	*B*	*t*	*p*	*B*	*t*	*p*
Constant	3.802	14.739	0.000**	3.864	15.383	0.000**	3.838	15.373	0.000**
Gender	–0.066	–0.914	0.361	–0.088	–1.252	0.212	–0.092	–1.324	0.187
Age	0.039	1.009	0.314	0.032	0.847	0.398	0.028	0.741	0.460
Education	–0.013	–0.206	0.837	–0.017	–0.274	0.784	–0.014	–0.240	0.810
ML	0.519	12.112	0.000**	0.372	6.805	0.000**	0.389	7.107	0.000**
IL				0.228	4.175	0.000**	0.234	4.321	0.000**
ML*IL							0.090	2.276	0.024*
*R* ^2^	0.350			0.388			0.399		
*F*	*F* (4,282) = 38.014, *p* = 0.000			*F* (5,281) = 35.669, *p* = 0.000			*F* (6,280) = 31.030, *p* = 0.000		

*p < 0.05, **p < 0.01.

### Simple slope test of the regulatory effect

**FIGURE 2:**
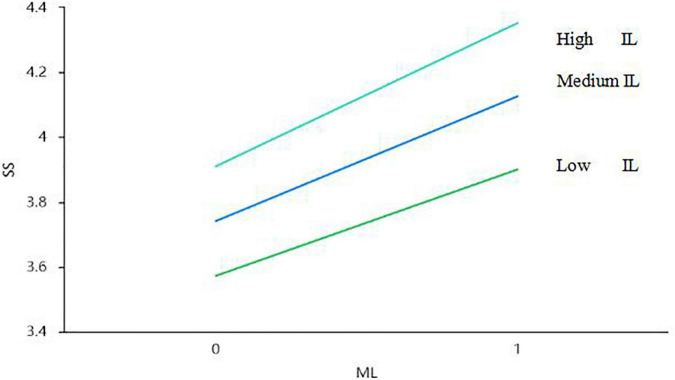
Moral leadership and psychological safety: the moderating effect of identification with leaders.

Figure 2 shows that IL positively moderates the relationship between ML and SS (that is, it enhances the positive relationship). Thus, H5 was confirmed.

## Discussion

### Theoretical implications

First, this study focuses on the relationship between ML and individuals’ innovation behavior in the domain of physical education. While many studies have focused on the impact of ML on individuals’ voice and work engagement, its impact on individuals’ innovation behavior is still in the early phase of research. Although previous studies have shown that ML is important in improving individuals’ innovation performance (Chen and Hou, 2016), there is a lack of investigation on the relationship between the two in the context of physical education. Existing research on PE teachers pays more attention to the impact of the working environment on basic teaching behavior, occupational fatigue, and other negative behaviors, without paying enough attention to the fact that positive innovation behavior is stimulated by leaders. Therefore, this study takes PE teachers as a study sample to expand the antecedents of PE teachers’ innovation behavior and further explore the impact of ML on PE teachers’ innovation behavior, which contributes to our understanding of the multiple interactive relationships between ML and different innovation behaviors on the individual level, which includes the relationship of ML–SS–IB and the relationship of ML–SS–EB. Furthermore, this paper explored how ML affects individuals’ innovation behaviors. For instance, ML matters in terms of both IB and EB.

Second, this study focuses on the relationship between ML and individual innovation behaviors, introduces individuals’ psychological safety as an intermediary variable, and explores how ML affects individuals’ psychology, it is worth emphasizing that ML has a significant tendency to further influence EB through the role of SS. Although previous studies have explored that ML indirectly stimulates team innovation behavior through SS, the study on team innovation behavior is focused on IB (Tu et al., 2019). Scholars pay more attention to the impact of the external environment on individual behavior in research on PE teachers, and rarely consider individuals’ cognition and psychology when studying the internal mechanisms in which ML motivates individual innovation behavior. Therefore, we have extended previous research by demonstrating that ML supports both internal and external innovation behavior of individuals in terms of forming SS, and this study enhances our understanding of the interplay between ML and different innovation behaviors of individuals.

Additionally, this study uses IL as a moderating variable and found that when employees’ IL, the relationship of ML–SS is strengthened, which would make the model more accurate. Theoretically, the current study mainly uses IL as a mediating variable (Khattak et al., 2022). but our research select IL as a moderating variable, further enriching the findings in this area and highlighting the need and urgency for further research on IL. Moreover, the introduction of this variable contributes new knowledge to the research on innovation teaching and the development of the sports industry to a certain extent.

### Managerial implications

The study offers insights for schools looking to cultivate and strengthen PE teachers’ innovation behaviors. For example, allows them to use the advantages of ethical human resource management to improve working environments. Furthermore, it provides a comprehensive and simple concept for leaders to apply appropriate leadership and cultivate the skills needed to ensure individuals’ SS and eliminate negative psychological factors that are not conducive to innovation. In this regard, ML is considered significant in the achievement of schools’ CSR practices, and SS and IL are considered significant in the achievement of efficient human resource management in schools. Besides, proposes suggestions for the career development of physical education talent in schools, who are more likely to be highly skilled composite personnel in the sports industry.

First, schools are encouraged to prioritize the recruitment and development of moral leaders, thereby improving the work environment for PE teachers and stimulating their IB and EB. Specifically, the candidates of leaders should be carefully examined in terms of characteristics, qualities, and thinking way during recruitment, so that those with outstanding ML can be selected. In addition, school administrators should advocate ML and establish a perfect system in which ethical standards and norms should be normalized in daily management. When leaders create a mutually respectful work environment in which followers feel safe and free to express their different views and come up with innovation achievements (Walumbwa and Schaubroeck, 2009), schools can adopt corresponding incentives like bonuses and praise to promote moral behavior.

Second, school administrators should ensure that PE teachers can perceive SS and work in an excellent environment in which PE teachers do not fear negative consequences from leaders or organization. Administrators can provide its resource support to ensure that individuals work in a safe environment. In ethical human resource management, leaders can encourage PE teachers to proactively participate in decision making and clarify organizational rules and regulations, provide moral support and material assistance to individuals facing difficulties to enhance cohesion between leaders and individuals, thereby improving individuals’ SS. Leaders should also consider balancing economic goals and ethical pursuits, understand the moral dilemmas and provide solutions and regular benefits to alleviate individuals’ sense of injustice and thus protect SS of individuals.

Finally, School administrators also need an emphasis on the role of individual IL. For example, school administrators can enhance IL with the focus on training of moral leaders and advocate them communicating with PE teachers in a mutual trust and respect atmosphere.

### Limitations and future research

This study helps organizations understand the impact of ML on individuals’ innovation behaviors. However, it still has some limitations and incredible room for improving. First, the research focuses on the teaching profession, so the results may be inapplicable to other professions, so future research could examine the impact of ML on individual innovation behavior in other professions. Second, the research is based on sample observations of Chinese PE teachers and doesn’t examine whether the impact of ML on individual innovation behavior varies by different cultures. Chinese culture emphasizes social harmony and collective thinking while western culture pay more attention to individual values realization. Therefore, future research should conduct comparative studies across national boundaries and cultures to test the applicability of conclusions. Finally, this study focused only on the mediating effect of SS between ML and innovation behavior, but there may be other mediating factors such as leader-member exchange (LMX). Therefore, these mediating variables can be parsed in future studies. In addition, future studies can adopt fuzzy set qualitative comparative analysis to improve the generalizability of the conclusions.

## Data availability statement

The original contributions presented in this study are included in the article/supplementary material, further inquiries can be directed to the corresponding authors.

## Author contributions

JC: conceptualization, methodology, software, and writing—reviewing and editing. WZ: writing—reviewing and editing and supervision. BJ: investigation, data curation, validation, and writing—original draft preparation. All authors contributed to the article and approved the submitted version.
